# Variability in Permanent Teeth Eruption in Children with Growth Hormone Deficiency and Idiopathic Short Stature

**DOI:** 10.3390/jcm14227896

**Published:** 2025-11-07

**Authors:** Natalia Torlińska-Walkowiak, Anna Sowińska, Katarzyna Anna Majewska, Andrzej Kędzia, Justyna Opydo-Szymaczek

**Affiliations:** 1 Department of Pediatric Dentistry, Poznan University of Medical Sciences, 60-812 Poznan, Poland; jopydo@ump.edu.pl; 2 Department of Computer Science and Statistics, Poznan University of Medical Sciences, 60-806 Poznan, Poland; ania@ump.edu.pl; 3 Department of Pediatric Diabetes, Auxology and Obesity, Poznan University of Medical Sciences, 60-572 Poznan, Poland; katarzynamajewska@ump.edu.pl (K.A.M.); akedzia@ump.edu.pl (A.K.)

**Keywords:** growth hormone, tooth eruption, growth hormone deficiency, short stature, tooth development

## Abstract

**Objectives**: Dental eruption is a complex process influenced by various factors, including endocrine factors as growth hormone (GH). The aim of this study was to assess differences in the advancement of tooth eruption between growth hormone-deficient (GHD) and idiopathic short-statured (ISS) children and a control group of children with normal growth patterns. **Methods**: A total of 156 children participated in this study: 78 patients with short stature (50 boys and 28 girls) and 78 healthy and age- and sex-matched control subjects. Each permanent tooth was classified according to its clinical eruption stage by one trained and calibrated dentist. **Results**: The mean age was 10.22 ± 2.42 years for the study and 10.15 ± 2.45 for the control group. In our study, we observed eruption delay during the early mixed dentition stage. A significant difference was found in the degree of eruption for all incisors and first permanent molars between the GHD before treatment group and the control group (*p* = 0.045). The difference was apparent at the initial stage of permanent tooth eruption, in the group of children who had not yet initiated growth hormone treatment. The eruption of remaining tooth groups did not differ significantly between the children with growth failure and the control group (*p* > 0.05). **Conclusions**: Our findings indicate that the delay in tooth eruption observed in short-statured children, particularly affecting the first permanent molars and incisors, may reflect the direct influence of growth hormone deficiency on early dental development. The clinical relevance of this finding underlines the importance of individualized dental care and careful timing of orthodontic assessments in short-statured patients, especially prior to the initiation of GH therapy.

## 1. Introduction

Dental eruption is a complex process influenced by various factors. Although it has been studied for decades, its course is not fully understood, particularly in certain disorders. Genetic and endocrine factors, systemic diseases, as well as local dental problems such as tooth–alveolar bone discrepancy, pathological conditions of primary teeth, and their early extractions can all modify tooth maturation and/or its subsequent emergence into the oral cavity [1,2]. Failure of eruption may occur as part of various craniofacial syndromes. In other cases, children may present with eruption disturbances associated with local factors such as cyst formation or previous traumatic injury [3]. When eruption disturbance is suspected, a thorough clinical examination complemented by appropriate imaging—such as radiography or cone-beam computed tomography (CBCT)—is essential to determine the location of the unerupted tooth and to identify potential absence or impaction [3].

One of the important endocrine factors, which is known to influence the development of the masticatory system and teeth, is growth hormone (GH) [4,5,6,7,8,9]. The GH/IGF axis is a significant regulator of bone formation, but in addition to its key role in bone physiology, it also exerts significant endocrine and autocrine/paracrine effects on mineralized tissues throughout life. Numerous publications have demonstrated links between the GH/IGF axis and IGF-binding proteins, as well as their corresponding receptors in dental tissues, including enamel, dentin, pulp, and periodontal ligament. GH and IGF-I induce the production of morphogenetic proteins 2 and 4 (BMP-2, BMP-4) and of the transforming growth factor-beta superfamily, promoting the differentiation of odontoblasts and ameloblasts [10,11,12].

In cases of GH deficiency, GH replacement therapy is widely used in young patients with short stature, and its influence on craniofacial growth has been extensively investigated [13,14,15]. It has been demonstrated that GH has a positive impact on the growth of the craniofacial skeleton: facial convexity decreases, while mandibular length and posterior facial height increase [5,13,15]. However, the relationship between GH, GH therapy, and dental morphology or eruption patterns remains unclear. One thing is certain—reduced dimensions of the maxilla and mandible often lead to dental crowding, as there is insufficient space to accommodate the wider permanent dentition [8,14,16].

Permanent teeth typically erupt following the exfoliation of their primary predecessors, with the exception of the permanent molars, which do not have primary counterparts.

In clinical studies, one key stage of eruption is usually analyzed: the eruptive phase, which is the period when the tooth moves from the bone through the soft tissue to appear in the oral cavity. Notably, permanent teeth erupt in two distinct stages: The first involves the eruption of the incisors and the first molars. After a pause of several years, the eruption of the remaining teeth follows. Davidopoulou and Chatzigianni concluded that tooth eruption remains unaffected by GH therapy, while other studies suggest some changes in dental age, with a reduced eruption delay observed in patients undergoing treatment [5,13,17].

Such discrepancies in the available evidence emphasize the need for further research, as limited data on dental age in short-statured and growth hormone-deficient children may complicate appropriate dental care. In this context, careful evaluation of two parameters: dental eruption timing and sequence is essential for diagnosis, orthodontic planning, and growth monitoring [18].

The aim of this study was to assess differences in the advancement of tooth eruption between growth hormone-deficient and idiopathic short-statured children and a control group of children with normal growth patterns. We hypothesized that children with growth failure would present with the delayed eruption of permanent dentition.

## 2. Materials and Methods

### 2.1. Examined Group and Sample Size

The incidence of growth hormone deficiency (GHD) is estimated at approximately 1 in 20,000 individuals [19,20,21]. In the Wielkopolska province, the population of children aged 7–17 years is about 400,000, which translates to an expected number of roughly 20 potential GHD cases in this age group. Based on this regional incidence, the initial sample size was calculated to ensure representativeness with a 5% margin of error and a 95% confidence level, yielding a minimal estimate of 20 participants. Given the rarity of GHD, all eligible patients meeting the inclusion criteria were invited to participate, as each examined case provides meaningful insight into the impact of growth hormone deficiency on craniofacial and dental development.

The observational study was conducted on patients undergoing clinical diagnosis or treatment for growth failure at a tertiary referral hospital from 2015 to 2024.

The diagnosis of growth hormone deficiency (GHD) or idiopathic short stature (ISS) was based on the following stimulation tests: glucagon (0.03 mg/kg of body weight), insulin (0.1 units/kg of body weight), or clonidine (100 mcg/m^2^). The decreased GH serum concentrations in two of those tests were required. According to the Polish national therapeutic program, the GH cut-off level was set at 10 ng/mL; therefore, children with peak GH levels below this threshold were diagnosed with GHD.

Standard medical assessments performed during diagnosis or GH therapy included X-Ray of the wrist in order to evaluate skeletal age according to Greulich & Pyle method [22,23]; serum IGF-1, TSH, FT4 levels, measurements of height using a stadiometer (SECA, Hamburg, Germany) with an accuracy of 1 mm, and body weight with an accuracy of 0.1 kg, and along with calculation of height standard deviation scores (hSDS) according to local Polish growth references, appropriate to age and gender.

The inclusion criteria were as follows: a confirmed diagnosis of short stature defined as hSDS below −2; completion of diagnostic process and a confirmed diagnosis of isolated growth hormone deficiency or idiopathic short stature; absence of any systemic diseases or any other conditions that may affect somatic growth; dentition during eruption and age between 5 and 14 years [24].

Exclusion criteria were defined as age outside the accepted range; a diagnosis of any additional diseases other than isolated GHD or idiopathic short stature; undernutrition; the presence of all permanent teeth in the occlusal plane at the time of dental examination, familial history of dental anomalies or examination of dental abnormalities that might affect tooth eruption; lack of parental or patients’ consent.

### 2.2. Control Group

For the control group, the following inclusion criteria were applied: generally healthy children with somatic growth appropriate for their chronological age and body height within the normal range. The control group was matched to the study group by age and sex. Every third child who met the inclusion criteria and was patients of the University Dental Centre attending routine dental check-ups were invited to participate in the study.

Exclusion criteria covered possible local factors that may be related to eruption abnormalities, such as traumas, missing or impacted teeth, supernumerary teeth, perinatal and postnatal problems.

All children were Caucasian, lived in the same geographic region, and were raised in similar socioeconomic conditions.

### 2.3. Clinical Eruption Assessment

Dental examinations were performed on all study participants by a single pediatric dentist (N.T.-W.). The examination focused on assessing the presence of permanent and primary teeth in the oral cavity and evaluating the eruption stage of permanent teeth.

Tooth emergence was recorded on an examination sheet by direct clinical inspection. Each permanent tooth was classified according to its clinical eruption stage, based on the system adapted from Carvalho et al. [25]:

- Stage 1: partially erupted crown—visible cusp(s) or incisal edge;
- Stage 2: less than half of the crown exposed;
- Stage 3: more than half of the crown exposed;
- Stage 4: full occlusion.

Calibration was conducted on ten children outside the study group to assess both intra- and inter-examiner reliability. The examiner demonstrated a high level of consistency with the trainer (J.O.-S.) and excellent reproducibility of repeated measurements (Kappa value > 0.80).

The results were presented with consideration of the medical diagnosis of short stature, distinguishing between ISS and GHD. Tooth eruption was depicted according to phases of eruption: the first phase of permanent tooth eruption (incisors, first molars) and second phase (premolars, canines and second molars).

The research protocol was approved by the Institutional Review Board of Poznan University of Medical Sciences, Poland (approval numbers: 785/15 and 777/23) and was conducted in accordance with the Declaration of Helsinki. Informed consent was obtained from all study participants and their legal guardians.

### 2.4. Statistical Analyses

The normality of distribution for continuous variables was assessed using the Shapiro–Wilk test. Quantitative data are presented as mean (standard deviation) for normally distributed variables or as median (interquartile range) for non-normally distributed variables. Student’s *t*-test and the Mann–Whitney U test were used to compare two groups of unpaired continuous data for normally and non-normally distributed variables, respectively. A *p*-value of <0.05 was considered statistically significant.

Statistical analyses were performed using PQStat Software (version 1.8.6.120) and MedCalc^®^ Statistical Software (version 22.014). The statistical power and effect size were calculated using the R software package (version 2025.09.1+401) and the pwr library.

## 3. Results

A total of 156 children participated in this study: 78 patients with short stature (50 boys and 28 girls) and 78 healthy, age- and sex-matched control subjects (50 boys and 28 girls).

The demographic characteristics of the study groups are presented in Table 1. Most children in the study group were in the mixed dentition stage of occlusal development (77%), which is characterized by the presence of both primary and permanent teeth in the oral cavity at the time of clinical examination. The permanent dentition stage, defined by the presence of only permanent teeth, was observed in 23% of the short stature group (including GHD and ISS patients) and 32% of the control group.

The mean age was 10.22 ± 2.42 years in the study group and 10.15 ± 2.45 years in the control group, with no statistically significant difference between them. Children during growth hormone substitution therapy were treated for an average of 3.12 ± 1.41 years.

Results regarding the stage of tooth eruption in the study and control groups are provided in Table 2. Data are presented separately for three subgroups within the study group: GHD before treatment, GHD during treatment, and ISS.

In the clinical examination, canines, premolars, and second permanent molars tended to emerge later in the GHD before treatment group compared to the age- and sex-matched control group; however, this difference was not statistically significant.

A significant difference was found in the degree of eruption of all incisors and first permanent molars between the GHD before treatment group and the control group (*p* = 0.045). Statistical significance was achieved (*p* = 0.045) with an effect size of d = 0.535 and a power of 82%. For the remaining comparisons, the post hoc calculated power ranged from 5% to 35%. In GHD during treatment group and ISS group, the difference in tooth eruption was not statistically significant (*p* > 0.05).

Results for males and females as compared with controls are presented in Table 3. Among girls with GHD before treatment, eruption was delayed compared to controls, particularly for incisors and first molars. In males before treatment, eruption was also delayed compared to controls for premolars, canines, and second molars, although the differences were not statistically significant.

Comparison between short stature girls and boys is shown in Table 4. There were no statistical significant differences between the sexes.

Further analysis of the eruption stages, comparing GHD before treatment with controls, for the GH cut-off set at 5 ng/mL and 7 ng/mL, is summarized in Table 5. The differences in the advancement of eruption were not statistically significant (*p* > 0.05) between groups.

## 4. Discussion

In order to obtain more detailed information about the dental status of short-statured patients, we decided to compare the degree of eruption of specific tooth groups between short-statured patients and their age-matched controls. To our knowledge, no previous studies have investigated these characteristics in children with GHD or ISS.

In our study, we included not only GHD patients (both those treated and untreated with growth hormone) but also a group of healthy short-statured children with normal endogenous GH secretion confirmed by endocrine testing and no other known cause of growth retardation, in order to identify potential changes strictly related to GH action.

The current study confirmed differences in tooth eruption between the study and control groups. Our hypothesis was supported by clinical examination, although it was evident for selected tooth groups. The difference was apparent at the initial stage of permanent tooth eruption, in the group of children who had not yet initiated growth hormone treatment. Our paper complements the knowledge about the possible effect of growth hormone level on the individual’s dental development, as it still remains unclear.

We also decided to investigate how the eruption progresses when considering the growth hormone level for GHD diagnosis from other European countries [26]. Analysis of the eruption stages comparing GHD patients before treatment with controls, for the GH cut-off set at 5 ng/mL and 7 ng/mL, did not reveal any differences in eruption. The most probable reason is the limited group of patients that fulfilled the criteria. It would be recommended to investigate this aspect in further research.

Primary and permanent tooth eruption refers to the movement of the tooth from its site of development in the alveolar bone into the oral cavity and towards the occlusal plane. This is a prolonged, multi-stage process that includes both pre-eruptive and post-eruptive phases [27]. The appearance of a dental crown in the oral cavity is only a clinical indicator that eruption is actively occurring. It’s important to note, however, that tooth eruption is one of the parameters of a child’s overall development. Several parameters of this process are important in assessing somatic development, primarily the timing of tooth eruption, but also the sequence of appearance of individual tooth groups [27].

The timing of dental eruption can vary widely across individuals [28,29,30] and may be affected by various pathological factors. A strong correlation has been demonstrated between eruption timing and dental maturity. Typically, primary and permanent teeth erupt when approximately two-thirds of their root length has developed [31]. The first phase of permanent tooth eruption, when both primary and permanent teeth are present in the oral cavity, begins with the eruption of teeth from the incisor and first molar groups [29]. The second transitional period starts with the clinical eruption of premolars or canines and continues until all canines, premolars, and second molars have emerged, usually by around twelve years of age. In the present study this order was maintained, which was visible in the degree of eruption of tooth crowns in patients with increasing age.

Clinical studies allow for the observation of differences in the timing of tooth eruption within different populations. The timing of individual teeth’s appearance in the oral cavity and the sequence of this phenomenon are most often recorded. The average eruption date is defined as the age at which a given tooth is present in the oral cavity in 50% of the studied population. An erupted tooth is considered any tooth visible in the oral cavity, regardless of the stage of eruption [27]. Furthermore, such studies are conducted on the entire group of children studied, regardless of their health status. This, of course, does not reflect the differences typical of individual diseases. Therefore, our analyses provide some additional information on the group of children with growth hormone deficiency.

Analyses of the timing of the eruption revealed that the first transitional period for Lithuanian children typically begins at a median age of 5 years and 10 months and ends at 7 years and 6 months. The second phase begins with the clinical eruption of the first upper premolars at approximately 9.5 years of age [32]. A study of Spanish children indicated that, in girls, permanent dentition typically begins to erupt at a mean age of 6.99 years and is usually complete by 12.45 years, while in boys, eruption occurs between 7.06 and 12.81 years of age [18]. Data for Polish children are comparable, ranging from 5.5 to 13.5 years of age [24]. Therefore, in our study we assumed that children eligible for inclusion should be between 5 and 14 years of age.

In both sexes, the commonly observed sequence of tooth eruption starts with the eruption of the lower central incisors and ends with the upper second molars [18,27,28]. However, recent Flemish studies demonstrated considerable variation, identifying 21 different eruption sequences in the maxilla and between 15 and 22 sequences in the mandible, each occurring with a prevalence of at least 1% [29]. The average tooth eruption period in the first stage of mixed dentition lasts approximately 2 years and 4 months, while the average duration of eruption for any tooth in the second stage is about 4 years and 2 months. These values are almost identical for both males and females. On average, first permanent molars and lower central incisors exhibit the shortest eruption periods, whereas second premolars have the longest [32]. It should be emphasized that, in our study, we observed eruption delay during the early mixed dentition stage. The degree of eruption of the central incisors, lateral incisors, and first permanent molars was significantly more advanced in healthy children compared to short-statured children of the same age. Importantly, this difference was evident only in patients before the initiation of GH therapy. It is plausible that GH levels in patients undergoing treatment, as well as in those with ISS, were sufficient to support normal eruption dynamics, although this aspect requires further investigation.

When the analysis was stratified by sex, a statistically significant delay was found only in girls with GHD before treatment. This suggests that GH deficiency may affect early-erupting teeth more noticeably in females, possibly because girls normally experience earlier dental maturation and eruption than boys [18,27,29]. Consequently, any growth-related delay may become more apparent in females, especially during the first transitional phase of mixed dentition. After GH therapy was initiated, no significant differences were observed, suggesting that hormonal supplementation effectively restores normal eruption timing.

However, these observations should be interpreted with caution, as the number of girls included in the study was small, reflecting the overall lower proportion of female patients receiving GH therapy in Poland and the generally higher prevalence of GHD among boys [8,33]. This imbalance may have limited the statistical power to detect subtler sex-related effects. Furthermore, our study was dependent on the timing of the patient’s report for diagnosis of growth disorder or a follow-up visit during treatment. This makes detecting statistically significant differences challenging in small patient groups observed over a limited time frame.

It is important to consider that the timing of tooth eruption, particularly for canines, premolars, and second permanent molars, exhibits greater individual variability.

The broad range of eruption sequences and timing observed in the second transitional phase, as reported by Leroy et al., may have reduced the sensitivity of our study to detect statistically significant differences in these later erupting teeth. In contrast, it might happen that the eruption of incisors and first permanent molars is generally more predictable and uniform across individuals, which could explain why delays were detectable specifically in this group. This inherent variability in the later erupting teeth suggests that larger cohorts and longitudinal studies may be necessary to fully assess the impact of growth hormone deficiency on the entire dentition [29].

The fact that a greater proportion of children in the study group remained in the mixed dentition stage suggests that short-statured patients may retain their primary teeth for a longer period compared to their healthy peers. This finding has clinical implications: while healthy children at this age may require only extractions, short-statured children may still need restorative treatment of primary teeth. Furthermore, premature extraction of primary teeth in short-statured children could exacerbate the tooth-bone discrepancy, leading to additional space deficiency for the eruption of permanent successors. Crowding of teeth is common in skeletal class II, and although we did not analyze this parameter [34], dental treatment in short-statured children should be carefully individualized, taking into account the child’s growth rate and developmental status. As in our previous study we demonstrated that bone age retardation is a more dominant feature than dental age delay in short-statured children with growth hormone deficiency [8], bone discrepancy may contribute to the development of malocclusions, as reduced jaw dimensions may be insufficient to accommodate relatively rapidly erupting teeth.

The slower rate of tooth eruption observed in patients from our study group may reflect general growth retardation, but it could also be partially associated with a space deficiency for erupting teeth. Tooth-alveolar discrepancy in short-statured children may contribute to altered eruption patterns. In fact, there is a significantly higher prevalence of dental crowding in the short-stature group (both GHD and ISS) compared to the healthy control group [8]. Orthodontic screening revealed that crowding was present in 57% of ISS patients and 53% of GHD patients before treatment, compared to only 22% of the control group (*p* = 0.027 and *p* = 0.021, respectively). Similar findings were reported also by Kjellberg et al. and Hodge et al., who found crowding prevalence in short-statured children to be 44% and 56%, respectively [14,16].

This study has several limitations that should be considered when interpreting the results.

First, the study was based on a one-time visual clinical examination, which involved assessing the presence of erupted teeth at a single time point. As a result, we were unable to monitor eruption over time, which could have provided valuable insight into the dynamics of the eruption process.

Additionally, it was also impossible to exclude all factors that could potentially influence tooth eruption. Radiographic examinations were not performed, as there were no clinical indications for exposing the children to X-rays. However, orthopantomographic images are important in setting the proper diagnosis of delayed tooth eruption. It might happen that the delay in eruption is caused by other than endocrinological issues.

Finally, since growth deficiency is a relatively rare condition, the study group was relatively small compared to typical eruption studies. Therefore, the role of growth hormone in dental development requires further longitudinal studies involving larger cohorts.

## 5. Conclusions

Our findings indicate that the delay in tooth eruption observed in short-statured children, particularly affecting the first permanent molars and incisors, may reflect the direct influence of growth hormone deficiency on early dental development. Interestingly, this delay was evident only in children who had not yet initiated growth hormone therapy, suggesting that timely GH supplementation may positively modulate the eruption process. This observation supports the hypothesis that growth hormone plays an active role in regulating the early stages of tooth emergence. The clinical relevance of this finding underlines the importance of individualized dental care and careful timing of orthodontic assessments in short-statured patients, especially prior to the initiation of GH therapy.

## Figures and Tables

**Table 1:** Characteristics of the study and the control group.

			Study Group N = 78	Control Group N = 78
Sex of participants	F	N (%)	28 (36%)	28 (36%)
	M	N (%)	50 (64%)	50 (64%)
Age	Years	Mean (SD)	10.22 (2.42)	10.15 (2.45)
Type of dentition	Primary and permanent	N (%)	60 (77%)	53 (68%)
	Permanent	N (%)	18 (23%)	25 (32%)
Duration of growth hormone substitution therapy	Years	Mean (SD)	3.12 (1.41)	n/a
Diagnosis	Idiopathic short stature	N (%)	29 (37%)	n/a
	GHD during treatment	N (%)	19 (24%)	n/a
	GHD before treatment	N (%)	30 (39%)	n/a
TSH		Mean (SD)	2.087 (0.81)	n/a
FT4		Me (Q1–Q3)	1.06 (0.97–1.16)	n/a
HSDS—height standard deviation scores		Me (Q1–Q3)/Mean (SD)	−2.35 (−2.77–2.077)	0.391 (0.96)

**Table 2:** Comparison of tooth eruption stages [Me(Q1-Q3)], between study and control groups (Mann–Whitney test) for the GH cut-off at 10 ng/mL.

Examined Teeth Groups	GHD Before Treatment N = 30 Me (Q1–Q3)	Control N = 30 Me (Q1–Q3)	*p*-Value	Effect Size	Power	GHD During Treatment N = 19 Me (Q1–Q3)	Control N = 19 Me (Q1–Q3)	*p*-Value	Effect Size	Power	Idiopathic Short Stature N = 29 Me (Q1–Q3)	Control N =29 Me (Q1–Q3)	*p*-Value	Effect Size	Power
Incisors and first molars	4 (2–4)	4 (4–4)	0.045	0.535	0.822	4 (4–4)	4 (4–4)	0.709	0.121	0.081	4 (3–4)	4 (3–4)	0.842	0.052	0.059
Premolars and canines	0 (0–4)	1.75 (0–4)	0.334	0.251	0.272	0 (0–4)	0.5 (0–4)	0.396	0.278	0.218	0 (0–4)	0 (0–4)	0.951	0.016	0.051
Premolars, canines and second molars	0 (0–3)	0.5 (0–4)	0.257	0.296	0.358	0 (0–4)	0 (0–4)	0.597	0.172	0.113	0 (0–3)	0 (0–4)	0.849	0.050	0.058
Age of patients	10.29 (7.92–12.25)	10.58 (7.67–12)	0.994	n/a	n/a	10.33 (9–12.83)	10 (8.5–13.5)	0.988	n/a	n/a	10.17 (7.42–11.92)	10.33 (7.25–12.00)	0.883	n/a	n/a

**Table 3:** Comparison of tooth eruption stages [Me (Q1–Q3)], between study and control groups, for male and female subgroups, (Mann–Whitney test) for the GH cut-off at 10 ng/mL.

Examined Teeth Groups	Sex of Patients	N	GHD Before Treatment Me (Q1–Q3)	Control Me (Q1–Q3)	*p*-Value	N	GHD During Treatment Me (Q1–Q3)	Control Me (Q1–Q3)	*p*-Value	N	Idiopathic Short Stature Me (Q1–Q3)	Control Me (Q1–Q3)	*p*-Value
Incisors and first molars	M	16	4 (2.75–4)	4 (4–4)	0.385	15	4 (4–4)	4 (4–4)	1.000	19	4 (2.25–4)	4 (1.5–4)	0.986
	F	14	4 (2.5–4)	4 (4–4)	0.038	4	4 (4–4)	4 (3.875–4)	0.453	10	4 (4–4)	4 (4–4)	0.871
Premolars and canines	M	16	0 (0–4)	1.75 (0–3.25)	0.521	15	0 (0–4)	3 (0–4)	0.345	19	0 (0–3.5)	0 (0–4)	0.791
	F	14	0 (0–3.75)	1.5 (0–4)	0.447	4	0 (0–0.75)	0 (0–0.125)	1.000	10	1 (0–3.5)	0 (0–3.75)	0.866
Premolars, canines and second molars	M	16	0 (0–2.5)	0.5 (0–3.25)	0.467	15	0 (0–4)	2 (0–4)	0.425	19	0 (0–3)	0 (0–4)	0.947
	F	14	0 (0–3)	0.5 (0–4)	0.367	4	0 (0–0.75)	0(0–0)	0.453	10	0 (0–3.5)	0 (0–2.625)	0.687

**Table 4:** Comparison of tooth eruption [Me (Q1–Q3)], between males and females for the GH cut-off at 10 ng/mL.

Examined Teeth Groups	GHD Before Treatment N = 14 Females	GHD Before Treatment N = 16 Males	*p*-Value	GHD During Treatment N = 4 Females	GHD During Treatment N = 15 Males	*p*-Value	ISS N = 10 Females	ISS N = 19 Males	*p*-Value
Incisors and first molars	4 (2.5–4)	4 (2.75–4)	0.979	4 (4–4)	4 (4–4)	0.511	4 (4–4)	4 (2.25–4)	0.481
Premolars and canines	0 (0–3.75)	0 (0–4)	0.941	0 (0–0.75)	0 (0–4)	0.483	1 (0–3.5)	0 (0–3.5)	0.702
Premolars, canines and second molars	0 (0–3)	0 (0–2.5)	0.960	0 (0–0.75)	0 (0–4)	0.483	0 (0–3)	0 (0–3)	0.938
Age of patients	9.87 (8.23–10.71)	10.37 (7.82–12.44)	0.560	9.88 (9.27–10.57)	10.33 (8.71–12.88)	0.802	10.63 (9.04–11.77)	10.17 (7.34–12.05)	0.945

**Table 5:** Comparison of tooth eruption for GHD before treatment group, when the GH cut-off is set at 5 ng/mL and 7 ng/mL.

Examined Teeth Groups	GH < 5 ng/mL N = 6 Me (Q1–Q3)	Control N = 6 Me (Q1–Q3)	*p*-Value	GH < 7 ng/mL N = 18 Me(Q1–Q3)/ Mean (SD)	Control N = 18 Me Q1–Q3)/ Mean (SD)	*p*-Value
Incisors and first molars	4 (4–4)	4 (4–4)	0.902 ^a^	4 (0–4)	4 (4–4)	0.097 ^a^
Premolars and canines	3.5 (0–4)	2.5 (0–4)	0.734 ^a^	0 (0–4)	2.75 (0–4)	0.439 ^a^
Premolars, canines and second molars	3.5 (0–3)	2.5 (0–4)	0.734 ^a^	0 (0–4)	1.75 (0–4)	0.371 ^a^

^a^ Mann–Whitney

## Data Availability

The data presented in this study are available upon request from the corresponding author.
